# Climate change implications for the distribution of the babesiosis and anaplasmosis tick vector, *Rhipicephalus* (*Boophilus*) *microplus*

**DOI:** 10.1186/s13567-020-00802-z

**Published:** 2020-06-17

**Authors:** Roberta Marques, Rodrigo F. Krüger, A. Townsend Peterson, Larissa F. de Melo, Natália Vicenzi, Daniel Jiménez-García

**Affiliations:** 1 Laboratório de Ecologia de Parasitos e Vetores, Programa de Pós Graduação em Microbiologia e Parasitologia, Departamento de Microbiologia e Parasitologia, Instituto de Biologia, Universidade Federal de Pelotas, Pelotas, RS Brazil; 2 Biodiversity Institute, University of Kansas, Lawrence, KS USA; 3 Centro de Agroecología y Ambiente, Instituto de Ciencias, Benemérita Universidad Autónoma de Puebla, Puebla, Puebla México

## Abstract

Climate change ranks among the most important issues globally, affecting geographic distributions of vectors and pathogens, and inducing losses in livestock production among many other damaging effects. We characterized the potential geographic distribution of the ticks *Rhipicephalus* (*Boophilus*) *microplus*, an important vector of babesiosis and anaplasmosis globally. We evaluated potential geographic shifts in suitability patterns for this species in two periods (2050 and 2070) and under two emissions scenarios (RCPs 4.5 and 8.5). Our results anticipate increases in suitability worldwide, particularly in the highest production areas for cattle. The Indo-Malayan region resulted in the highest cattle exposure under both climate change projections (2050), with increases in suitability of > 30%. This study illustrates how ecological niche modeling can be used to explore probable effects of climate change on disease vectors, and the possible consequences on economic dimensions.

## Introduction

*Rhipicephalus* (*Boophilus*) *microplus* is the most important tick in transmission of bovine parasitic diseases around the world [1]. The principal hosts for this species are cattle, but interactions have been shown with buffalo, horses, donkeys, dogs, deer, sheep, and goats [2–4]. High incidence of this tick is associated with economic losses, particularly in cattle [5]. This tick is responsible for transmission of the protozoa *Babesia bovis* and *B. bigemina*, and the bacterium *Anaplasma marginale*, which are the main pathogens of bovine babesiosis and anaplasmosis, respectively [6–8]. These diseases induce extreme emaciation in livestock, culminating in death.

Losses of US$13–18 billion are caused by these pathogens globally each year [9, 10]. Developing countries have seen the strongest consequences caused by *R*. *microplus* (e.g. in South America; [5, 11]). Such countries generally lack effective control mechanisms for the tick, so that economic losses are exacerbated and livestock production is reduced markedly [12]. Estimates regarding milk losses caused by this tick species are 90.2 L per cow yearly; this reduction and losses in milk products generate production drops of around US$922 million yearly [13]. Cattle at high risk regarding this tick are valued at US$3 billion annually in Brazil alone [5], one of the most important developing countries in terms of livestock, responsible for exporting ~ 43 million tons of milk and meat [14].

Population growth and establishment are related to the following: (1) historical contingencies and geographic barriers [15]; (2) biological factors such as host availability, competition; and (3) environmental conditions such as temperature and humidity [16, 17]. Future changes in climate include modifications of temperature and precipitation regimes; these environmental factors are crucial in delimiting species distributions and determining the success of population establishment [18]. Changes in these factors may modify the life cycle, abundance, and distribution potential for *R. microplus* [19, 20] and other disease vectors or species with importance in human and animal health [21–23]. Small changes at local levels can increase the risk of pathogen transmission [24]. Climate change generates a series of biological modifications in vector biology and consequently in pathogen incidence, which may lead to shifts in disease distributions [25].

Ecological niche models (ENM) are commonly used for understanding species’ potential geographic distributions under different scenarios of environmental change [26–28], under Grinnell’s niche concept [29]. The hypothesis of environmental factors being crucial in determining distributions of vectors and pathogens is supported amply by empirical studies [21, 22, 30–34]. However, many based-ENM studies of related disease arthropods have not used environmental variables related directly to the physiology of these species instead relying on general climate datasets that are easily available. In this paper, we assess possible potential areas for *R. microplus* under present-day and future climate conditions for two greenhouse gas emissions scenarios (RCP 4.5 and RCP 8.5) in two time periods (2050 and 2070), including a variable known to be crucial to the physiology of this species. We evaluate potential hotspots for this species and their coincidence with livestock concentrations to determine the most important risk areas under climate change for bovine parasitic diseases transmitted by this tick species.

## Materials and methods

### Occurrence data

Occurrences were obtained from two different sources: (1) published data based on searches of different databases (Web of Science, Scopus, and Google Scholar); we used the keywords “*Rhipicephalus*”, “*Rhipicephalus microplus*”, “*Boophilus microplus*” and “*Rhipicephalus* (*B.*)*. microplus*”. We also obtained (2) data available on biodiversity information platforms: Global Biodiversity Information Facility [35], SpeciesLink [36], and VectorMap [37].

**Figure 1:**
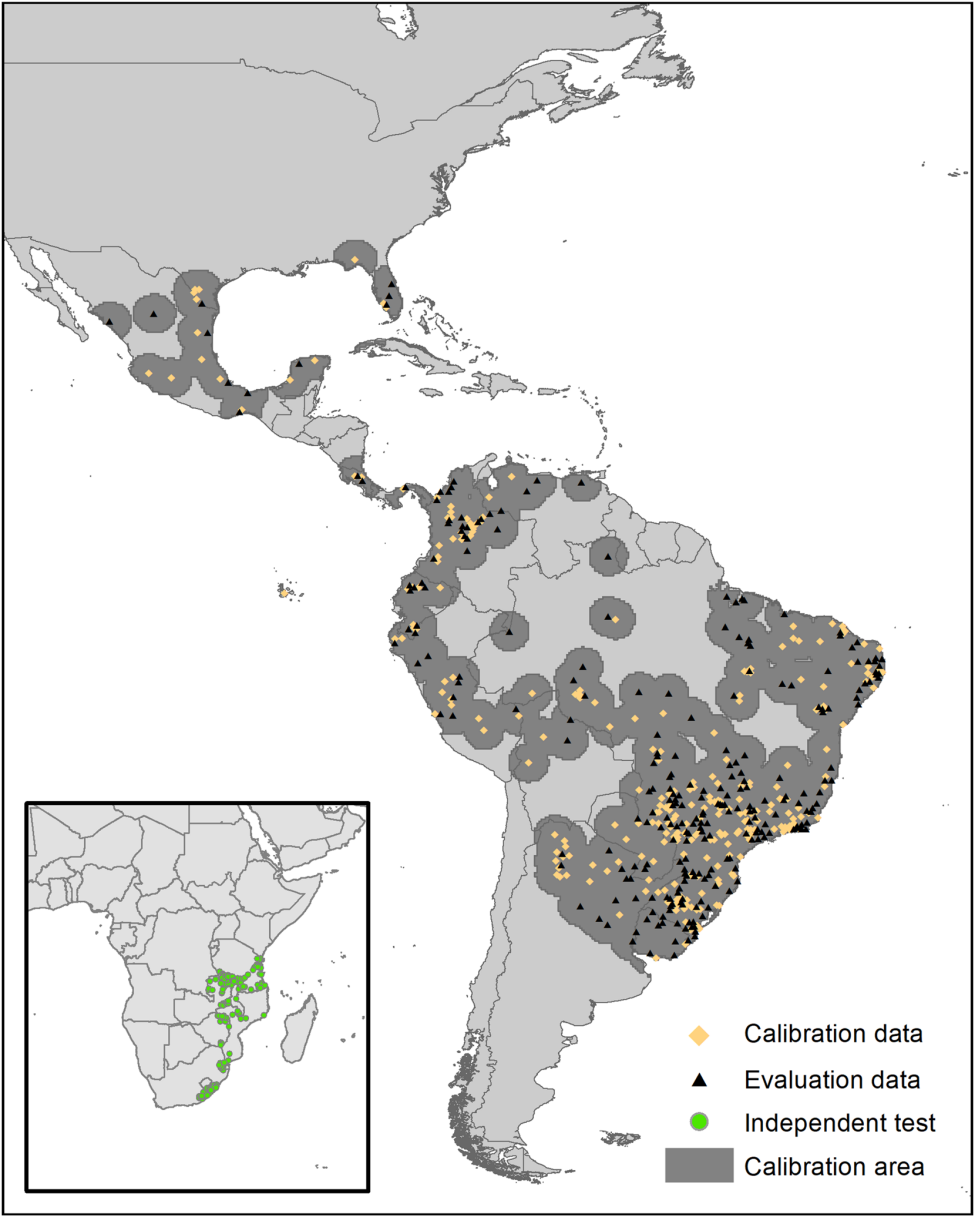
**Calibration area and the known distribution of*Rhipicephalus*(*Boophilus*)*microplus*(points).** Occurrence records used in model calibration (test and training) are shown; occurrences in Africa were used as independent data to evaluate the accuracy of model transfer worldwide.

Data were collected for the period 1970–2018. Data lacking georeferencing (obtained mainly from published papers) were assigned coordinates via searches in Google Earth. We reduced biasing effects of spatial autocorrelation in occurrence data using a distance filter of 22 km in the spThin R package [38]. We chose a random 50% of the occurrence data for calibrating models, and used the remaining 50% to evaluate the models. Our initial 1487 occurrences for *R. microplus* in America reduced to 531 with spatial filtering (Figure 1). We also considered occurrence data for this species from Africa [39, 40]; these 145 African occurrences were used as independent evaluation data, with the same spatial filter, for an additional model evaluation.

### Environmental data

We used 15 bioclimatic variables from WorldClim version 1.4 (Table 1) [41], excluding four variables known to include spatial artefacts [42]. WorldClim variables were derived from climatic data for 1950–2000. To add a variable known to be important to the physiology of this species [43], we obtained relative humidity (RH) from the Coupled Model Intercomparison Project [44], which we downscaled by the Delta Method, commonly used in climate data [45]. To summarize future conditions, we used outputs from 20 general circulation models (GCM) available from Climate Change, Agriculture and Food Security [46] (Additional file 1). We used two greenhouse gas emissions scenarios (RCP 4.5 and RCP 8.5) for two time periods (2050 and 2070) to explore model-to-model variation. The environmental data were used at a spatial resolution of 0.2° (~ 22 km) under both present-day and future conditions. Dimensionality was reduced by calculating Pearson correlations over the entire calibration area, removing one from each pair of variables with correlations ≥ 0.80. Uncorrelated variables were according to different variable sets: we used all possible variable combinations (120 sets) for model calibration and evaluation [47]. Variables used as candidates for inclusion in models therefore included annual mean temperature, temperature seasonality, minimum temperature during the coldest month, annual precipitation, precipitation during the driest month, precipitation seasonality, and relative humidity.

**Table 1:** **Climate variables used in ecological niche modeling of current and future potential distributions of*Rhipicephalus*(*Boophilus*)*microplus***

Acronym	Description	% variable contribution
Bio1^a^	Annual mean temperature	26.6
Bio2	Mean diurnal range	
Bio3	Isothermality	
Bio4^a^	Temperature seasonality	21.4
Bio5	Maximum temperature of warmest month	
Bio6	Minimum temperature of coldest month	
Bio7	Temperature annual range	
Bio8^b^	Mean temperature of wettest quarter	
Bio9^b^	Mean temperature of driest quarter	
Bio10	Mean temperature of warmest quarter	
Bio11	Mean temperature of coldest quarter	
Bio12^a^	Annual precipitation	14.6
Bio13	Precipitation of wettest month	
Bio14^a^	Precipitation of driest month	19.2
Bio15	Precipitation seasonality	
Bio16	Precipitation of wettest quarter	
Bio17	Precipitation of driest quarter	
Bio18^b^	Precipitation of warmest quarter	
Bio19^b^	Precipitation of coldest quarter	
RH^a^	Relative humidity	18.1

Bioclimatic variables from the WorldClim data archive (version 1.4; Hijmans et al. [41]) and relative humidity [44] were used for modeling. Five variables^a^ were selected for modelling via our model selection processing [47].

^b^Variables excluded because they have unrealistic spatial artefacts [42].

### Model calibration and evaluation

Models were calibrated with the kuenm R package, running [48] Maxent 3.4.1 [49] and using model selection approaches [50]. We used significance, performance (omission rate), and model complexity to choose optimal parameter settings from among candidate models. All possible combinations of linear (l), quadratic (q), product (p), threshold (t), and hinge (h) feature types were tested, as were different regularization multiplier values (0.1, 0.3, 0.5, 0.7, 1, 2, 3, 5, 7, and 10). Models are built using all possible combinations of the seven environmental variables. Hence, we explored a total of 5400 candidate models. Significance testing was via partial ROC [51], with acceptable omission error of *E* = 0.05. Finally, we evaluated the model complexity using the Akaike information criterion with a correction for small sample size (AICc), via a code derived from Warren et al. [50].

We used the accessible area concept as a means of choosing the area over which to calibrate our models [15, 52], using a buffer of 200 km around occurrence data as a proxy to this area (Figure 1). Final models were summarized as the median of 10 bootstrap replicates of the model corresponding to the best model parameter set, and transferred to future conditions worldwide. We used the kuenm package [48] both to evaluate final models and to transfer models to future conditions. Areas presenting extrapolative conditions were identified with a MOP analysis [53], comparing conditions between calibration and transfer areas across the 20 GCM × 2 RCPs × 2 time periods that made up our future scenarios.

Model transfers were summarized and simplified via a binarization process. Median model transfers were binarized using an acceptable omission rate of *E* = 0.05. Binary maps were used to determine climate uncertainty in the models, which we summarized as agreement among multiple scenarios between present and future to determine areas of stability. We used a threshold of ≥ 60% (12) of agreement among GCM as a relatively clear signal of presence or absence of suitable conditions. Finally, to provide an additional model evaluation, we used occurrence data from the African range as an evaluation of the model’s predictive ability. We obtained cattle abundance data from the Food and Agriculture Organization of the United Nations (FAO) [54] and Robinson et al. [55] to evaluate implications of future changes in the range of the tick for cattle production. We used different categories (cattle abundances: Additional file 2) to evaluate possible impacts of the tick under future projections. We evaluated the highest potential range expansion of the tick with regards to the different world cattle production areas.

## Results

We evaluated 5400 candidate models; 3687 of these models were statistically significant (*P *< 0.05), of which 1348 showed good performance (i.e., omission error ≤ 0.05); however, a single model was selected on the basis of low complexity (AICc = 10,398.67), in that the difference in AICc between it and the next best model was large (26.4786). The best model included all feature types (lqpth: Linear, Quadratic, Product, Threshold and Hinge) with a regularization parameter of 1. Variables selected were annual mean temperature, temperature seasonality, annual precipitation, precipitation of driest month, and relative humidity (Table 1). The biggest relative contribution of environmental variables to the model was from annual mean temperature (26.6%), whereas the smallest contribution was from annual precipitation (14.6%; Table 1). Our independent evaluation (i.e., predicting the African distribution) was significantly different from random predictions according to the pROC evaluation (*P* < 0.001). Omission rate was 7.5%, with only 11 failures out of 145 evaluation points.

**Figure 2:**
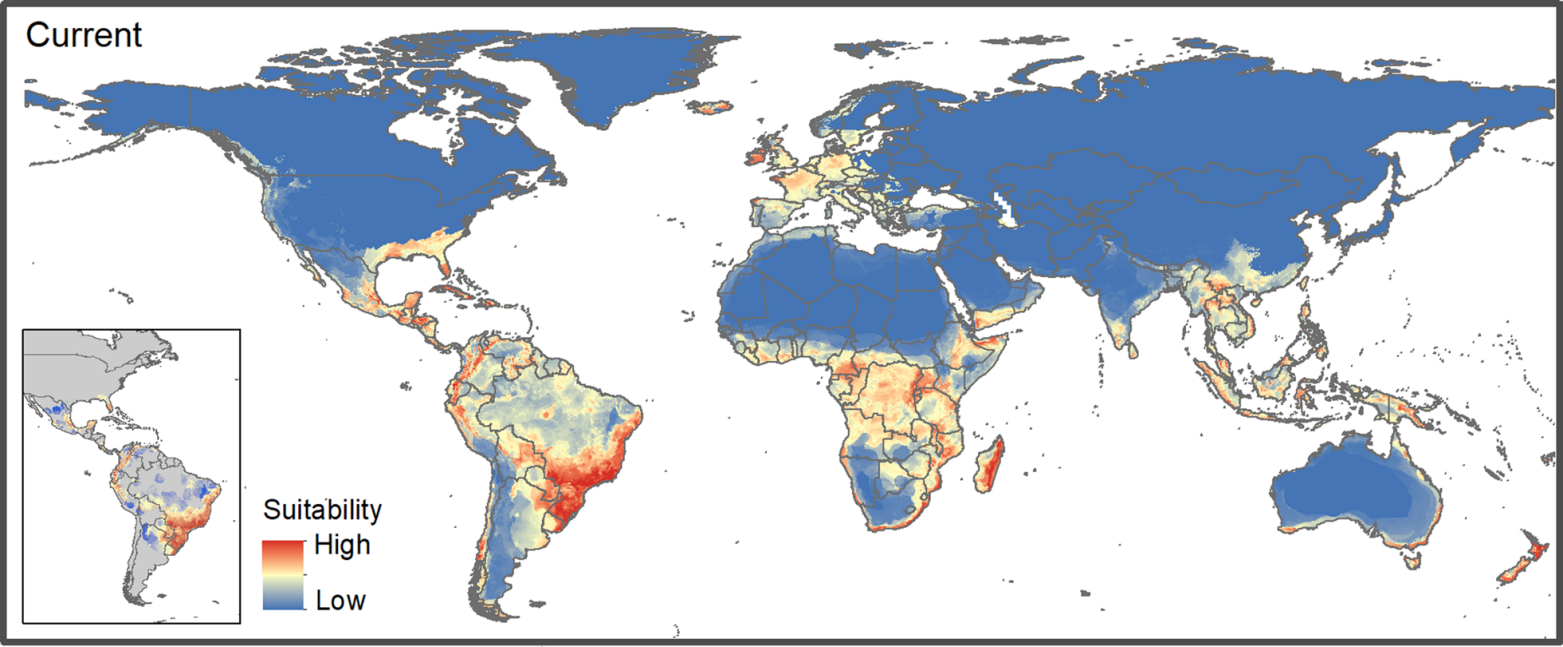
**Present-day suitability for*Rhipicephalus*(*Boophilus*)*microplus*according to the best ecological niche model, under current conditions and calibration area (detail).**

In the Americas, the present-day model shows high suitability for *R. microplus* in North and South America: in Brazil (central, western, southern), Uruguay (northern), Argentina (northern, eastern), and across Central America, Mexico, and the southern USA (Figure 2). Models also indicate high suitability across much of sub-Saharan Africa, except for the interior of South Africa and Botswana. Western Europe, Southeast Asia, and coastal parts of Australia also had high suitability (Figure 2), especially inside the calibration area.

**Figure 3:**
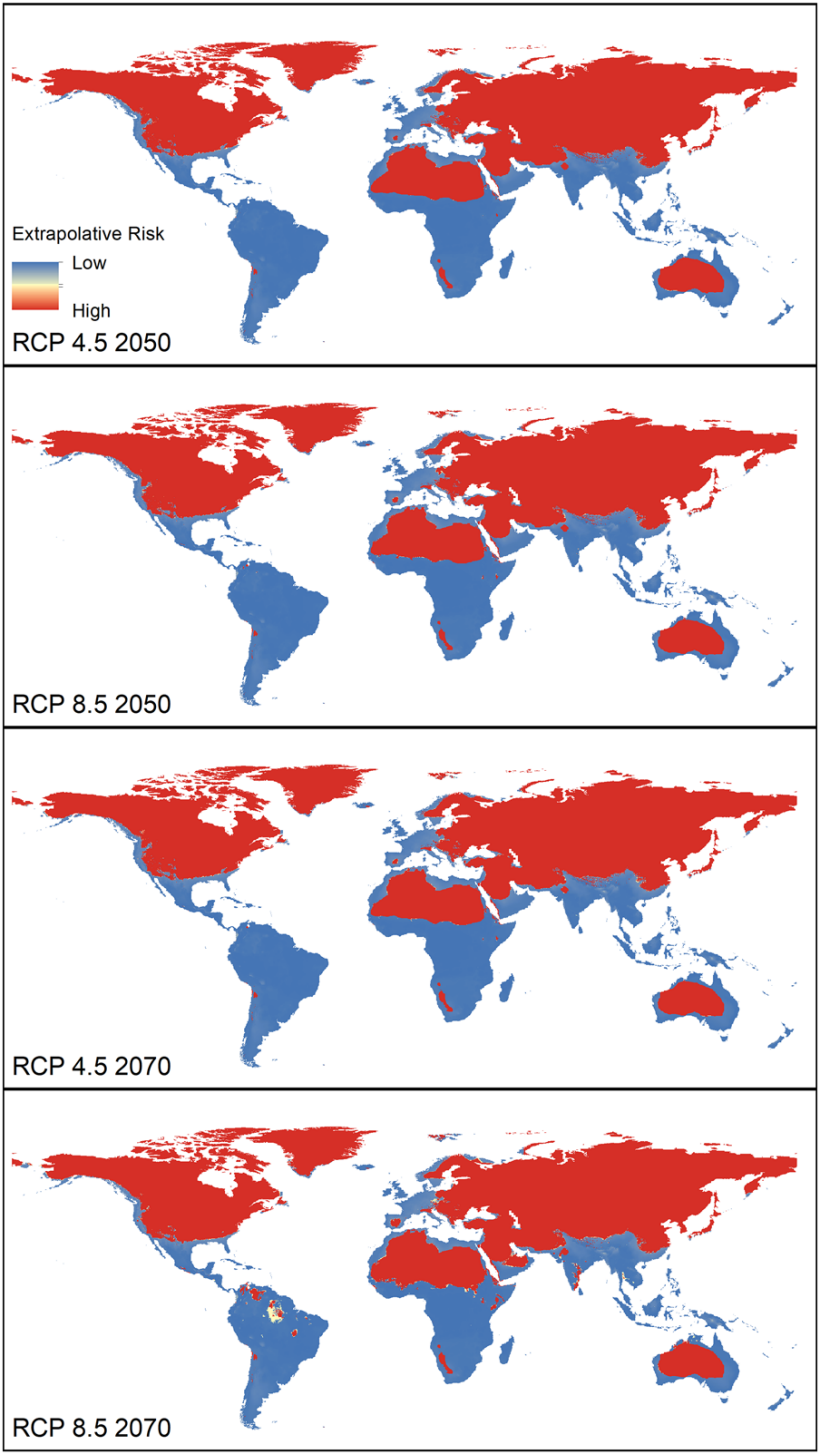
**MOP analysis of extrapolation risk from the calibration area under current conditions to the whole world under future conditions.** Blue values represent strict extrapolative areas. Red values represent levels of similarity between the calibration area and the different RCPs scenarios of projections.

Model transfers to future conditions (Additional files 3, 4, 5 and 6) show high stability of suitability in currently suitable regions, and increases of suitability in the Neotropics (Argentina, Brazil, Colombia, Venezuela), Eurasia (northern and eastern Europe; Indo-Malayan Region (India, Bhutan, Nepal, Myanmar, China), North America (Mexico, southeastern USA), and including Afrotropics (West Africa, Sudan, South Sudan, Chad). Under a moderate climate change scenario (RCP 4.5) we noted increases in suitability with low uncertainty by 2050 and 2070 (Additional files 3 and 4); some areas of South America (e.g., Amazonas state in Brazil) show increased suitability. Under the high-emissions scenario (RCP 8.5), in 2050, increases in suitability were broadly distributed in the Nearctic, Neotropics, Palearctic, Afrotropics, and the Indo-Malayan region (Additional files 3 and 5). For 2070 (Additional files 4 and 6), increases in suitability were in much the same regions. The areas that presented extrapolation risk from the calibration area are shown in Figure 3.

Our models show high suitability in the present day, and increases in suitability in the future, in places with the highest abundances of cattle around the world (Tables 2 and 3, Additional file 2). Low cattle abundances (0 to 1 individuals/10 km^2^) were the areas most likely to see increases in suitability for the tick, with a possible increase of ~ 21% in the world for this category (Tables 2 and 3); the Palearctic, Neotropical, and Indo-Malayan regions were those most likely to see increases in suitability. Highest cattle abundances (> 100 individuals/10 km^2^) in the Indo-Malayan region were projected to see suitability increases of ~ 34% in 2050 and ~ 16% in 2070 (Tables 2 and 3). All study regions show increases in suitability (~ 1% to ~ 134%); major changes were noted in the Nearctic Region (Tables 2 and 3).

**Table 2:** **Proportion of modification of*R***. ***microplus*suitability to climate change scenarios according to zoogeographic regions and livestock abundance categories worldwide [**54, 55**] for 2050 scenarios**

Scenarios	Regions	Cattle abundance (individuals/10 km^2^)							
		0–1	1–5	5–10	10–20	20–50	50–100	> 100	All categories (average)
RCP 4.5	Afrotropic	1.57	5.78	6.47	6.23	5.86	6.93	7.75	5.80
	Australasia	6.26	6.10	0.91	1.41	9.94	7.81	3.99	5.20
	Indo-Malayan	3.02	2.82	3.55	3.27	5.64	*11.44*	*31.55*	8.76
	Nearctic	*16.09*	3.62	2.87	4.52	4.40	2.46	*25.48*	8.49
	Neotropic	9.42	5.71	4.87	5.61	5.70	4.44	8.95	6.39
	Palearctic	9.05	2.83	3.22	4.32	3.13	0.79	0.49	3.40
RCP 8.5	Afrotropic	0.60	1.96	1.33	2.19	1.98	1.23	1.72	1.57
	Australasia	1.53	0.50	0.06	0.16	0.61	0.00	0.00	0.41
	Indo-Malayan	3.39	2.34	4.24	4.36	6.04	6.54	6.28	4.74
	Nearctic	*33.12*	2.50	1.90	1.62	5.65	*32.89*	*134.87*	30.36
	Neotropic	2.69	2.68	2.40	2.33	1.90	1.29	0.59	1.99
	Palearctic	*22.88*	*26.74*	*12.78*	*10.35*	6.61	4.80	1.78	12.28
Both	Afrotropic	4.58	*10.21*	*14.19*	*11.24*	7.80	7.77	9.97	9.39
	Australasia	*13.65*	5.97	8.18	7.26	9.26	7.92	2.91	7.88
	Indo-Malayan	*25.17*	*12.77*	*11.14*	*11.91*	*18.24*	*20.53*	*34.57*	19.19
	Nearctic	*50.34*	4.25	2.50	1.68	2.35	8.62	3.01	10.39
	Neotropic	*29.87*	*13.68*	*12.14*	9.71	7.82	5.44	8.87	12.50
	Palearctic	*32.03*	*40.84*	*18.65*	*15.71*	*13.09*	8.95	4.51	19.11

Values with increases greater than 10% are shown in italics.

**Table 3:** **Proportional modifications of*Rhipicephalus*(*Boophilus*)*microplus*suitability to climate change scenarios divided by zoogeographic regions and livestock abundance categories worldwide [**54, 55**] for 2070 scenarios**

Scenarios	Regions	Cattle abundance (individuals/10 km^2^)							
		0–1	1–5	5–10	10–20	20–50	50–100	> 100	All categories (average)
RCP 4.5	Afrotropics	3.23	8.86	*10.68*	*10.75*	9.04	5.10	1.96	7.09
	Australasia	7.09	7.30	2.97	3.51	9.65	0.90	2.60	4.86
	Indo-Malayan	5.73	6.43	8.60	7.84	*11.35*	*16.99*	*47.84*	14.97
	Nearctic	*11.66*	2.19	1.81	2.03	1.22	0.82	0.00	2.82
	Neotropics	*19.67*	*11.25*	*11.39*	9.47	8.88	6.95	*11.44*	11.29
	Palearctic	6.60	6.31	5.16	5.63	3.19	1.64	0.48	4.15
RCP 8.5	Afrotropics	0.09	0.81	0.50	0.75	1.16	4.03	5.97	1.90
	Australasia	5.19	1.71	0.47	0.26	0.32	0.76	0.10	1.26
	Indo-Malayan	6.03	1.50	1.94	2.17	2.51	4.81	3.47	3.20
	Nearctic	*86.15*	2.25	1.11	0.78	1.73	4.97	4.03	14.43
	Neotropics	0.95	2.02	1.33	1.45	1.76	1.06	0.09	1.24
	Palearctic	*39.17*	*38.77*	*26.97*	*18.92*	*13.40*	4.01	2.68	20.56
Both	Afrotropics	2.05	3.96	6.83	4.66	3.68	6.87	*11.02*	5.58
	Australasia	*10.49*	3.33	8.50	7.70	*13.94*	*10.34*	7.29	8.80
	Indo-Malayan	*18.37*	*10.01*	9.32	9.93	*15.52*	*13.45*	*16.74*	13.33
	Nearctic	*77.68*	4.88	1.88	1.39	1.23	5.50	3.01	13.65
	Neotropics	*14.48*	5.44	5.14	4.15	3.31	1.91	3.70	5.45
	Palearctic	*38.34*	*42.56*	*21.47*	*23.21*	*18.73*	*13.60*	6.33	23.46

Values ≥ 10% are shown in italics.

## Discussion

Babesiosis and anaplasmosis may be related to the same environmental factors as *R*. *microplus* because they depend on this tick as a main vector [6–8]. Different wild animals serve as hosts for this tick; the best-studied is the white-tailed deer *Odocoileus virginianus* [3]: livestock reservoirs of these pathogens include goats [4]. Host species are important for dispersal, although this tick is not a specialized ectoparasite; this generalist habit facilitates dispersal by wild and livestock animals [6–8].

The potential distribution of *R. microplus* is related to a diverse suite of ecological and environmental factors around the world [1, 17, 19, 20, 56–60]. Moreover, in order for the tick populations to spread in a region, individuals must first be introduced, then go through a phase of adaptation to the local hosts and then reach a population density that allows mating and reproductive success of adults [1, 31, 58]. Complex relationships with other species, especially wild hosts, are particularly relevant for individual dispersal and population occurrence in suitable environments [3, 61]. Our models show suitable areas in Europe and Asia, places without species present; however, the environmental conditions correspond to species establishment. Climate factors affect the ticks life cycle and geographic distribution [62, 63]. The most important factors identified in our model construction were annual mean temperature, precipitation seasonality, and relative humidity [17].

Climate seasonality is an important factor in the *R. microplus* life cycle; variation in this factor influence the number of generations (three to four per year), increasing the population size and potentially facilitating dispersal [62, 63]. We found important environmental variables similar to those identified in field analyses of this species [17]. Annual mean temperature, seasonality in precipitation, and variables derived from humidity were crucial to these models [17]; however, no previous study has used relative humidity in model construction, which is a fundamental variable affecting this species’ development [64, 65].

Currently, *R. microplus* does not occur in some regions that our models signaled as suitable, which can be explained by several factors; for example: (1) some trophic activities or other biological interactions that reduce the possibility of occurrence in Europe, Australia, and parts of northern Asia. (2) Historical conditions in particular, major dispersal barriers, decrease the access of this species to Europe, Australia, and northern Asia. In addition, for the species to be introduced into these areas, it is important to consider the number of individuals required to overcome demographic limitations and produce permanent populations, sufficient host density to support tick populations, and immune responses of the host [17].

Economic losses in cattle are generated by tick infestations in cattle herds around the world. Countries with high cattle populations have experienced significant losses in meat production (e.g., Brazil, with US$3.4 billion per year [5]; Tanzania, with US$364 million per year [66]; and Mexico, with US$573 million per year [67]). Thirty-eight percent of the world’s cattle population is located in India; babesiosis is a common disease there, driving important economic losses [68]; indeed, ~ 4% of cattle in northeastern India died from this disease [69]. Our results anticipate increases in suitability for the tick, particularly in northern India, potentially increasing losses under future climates (Tables 2 and 3, Figures 2 and 3, Additional files 3 and 4). The Indo-Malayan regions include several countries with high potential cattle exposure showing increases in tick suitability (Additional files 3 and 4) and high abundances of cattle, particularly south India, Sri Lanka, Myanmar and Thailand (Tables 2 and 3, Additional file 2). Increases in suitability for the tick in West Africa (Côte d’Ivoire, Benin), increases cattle exposure to babesiosis and anaplasmosis [31]; of particular note is that importation of Brazilian cattle in these countries creates a situation optimal for tick establishment a situation confirmed by independent models from De Clercq et al. [31].

Changes in the potential distribution of this tick in relation to climate change have been discussed and documented in previous studies [17, 19, 56]. However, our work used an uncertainty evaluation that explored 20 GCM, two greenhouse gas emission scenarios, and two time periods (Additional files 3 and 4): we also used independent data for model testing (from Africa) and incorporated novel data on relative humidity. Our model is strongly consistent and accurate (Additional files 3, 4, 5), but has two strong limitations. (1) We did not include biotic factors (interactions) in the model, especially because this species requires a host; however, given the broad host range of this tick, we did not have any logical means of assessing all the different possible hosts in the world. (2) We did not consider relationships between numbers of ticks and numbers of cattle in different regions of the world. However, our models provide a view of suitability for the tick under future conditions, which we translate into metrics of cattle exposure for different cattle populations worldwide. The results were consistent with future potential increases of this invasive species around the world, despite the biotic factors not evaluated in our models (e.g. vegetation, host availability and biological interactions).

## Supplementary information

**Additional file 1. General circulation models used in ecological niche modeling projections in RCP 4.5 and RCP 8.5 for 2050 and 2070.**

**Additional file 2. Cattle abundances categorized from FAO and Robinson et al. [**54**] from different zoogeographic regions in the world to evaluate*Rhipicephalus*(*Boophilus*)*microplus*suitability in each of them under future climate change and present-day scenarios.** Abundances are represented by number of individual heads of cattle per 10 km^2^.

**Additional file 3. Current and potential future distributions of*Rhipicephalus*(*Boophilus*)*microplus*for two emissions scenarios (top, RCP 4.5; bottom, RCP 8.5) in 2050.** Dark blue: areas predicted to be suitable in present-day and with a strong chance of suitability in the future (> 12 GCM). Light blue: areas predicted to be suitable in present-day but with reduced probability of presence in the future (< 12 GCM). Red: areas unsuitable in the present-day, but with a strong chance of suitability in the future (> 12 GCMs). Pink: areas predicted to be unsuitable in present-day but have slight chance of suitability in the future (< 12 GCM). White: areas unsuitable in both present-day and future scenarios.

**Additional file 4. Current and potential future distributions of*Rhipicephalus*(*Boophilus*)*microplus*for two emissions scenarios (top, RCP 4.5; bottom, RCP 8.5) in 2070.** Dark blue: areas predicted to be suitable in present-day and with a strong chance of suitability in the future (> 12 GCM). Light blue: areas predicted to be suitable in present-day but with reduced probability of presence in the future (< 12 GCM). Red: areas unsuitable in the present-day, but with a strong chance of suitability in the future (> 12 GCM). Pink: areas predicted to be unsuitable in present-day but with a slight chance of suitability in the future (< 12 GCM). White: areas unsuitable in both present-day and future scenarios.

**Additional file 5. Suitability for*Rhipicephalus*(*Boophilus*)*microplus*in 2050 under RCP 4.5 and 8.5 according to the best ecological niche model.**

**Additional file 6. Suitability for*Rhipicephalus*(*Boophilus*)*microplus*in 2070 under RCP 4.5 and 8.5 according to the best ecological niche model.**
