# Supplementary material for: Climate change implications for the distribution of the babesiosis and anaplasmosis tick vector, Rhipicephalus (Boophilus) microplus
Source: Vet Res. 2020 Jun 17;51:81. doi: 10.1186/s13567-020-00802-z (PMC7298856; doi:10.1186/s13567-020-00802-z)
Supplement: Supplementary file 1 — Additional file 1. General circulation models used in ecological niche modeling projections in RCP 4.5 and RCP 8.5 for 2050 and 2070. [file 13567_2020_802_MOESM1_ESM.doc]

**Additional file 1 General circulation models used in ecological niche modeling projections in RCP 4.5 and RCP 8.5 for 2050 and 2070.**

| **General circulation model acronym** | **Institution** |
| --- | --- |
| bnu_esm | Beijing Normal University Earth System Model |
| cesm1_bcg | National Center for Atmospheric Research, USA |
| cesm1_cam5 | National Center for Atmospheric Research, USA |
| csiro_access1_3 | Commonwealth Scientific and Industrial Research Organization (CSIRO) and Bureau of Meteorology (BOM), Australia |
| csiro_access1 | Commonwealth Scientific and Industrial Research Organization (CSIRO) and Bureau of Meteorology (BOM), Australia |
| gfdl_cm3 | NOAA Geophysical Fluid Dynamics Laboratory |
| gfdl_esm2g | NOAA Geophysical Fluid Dynamics Laboratory |
| gfdl_esm2m | NOAA Geophysical Fluid Dynamics Laboratory |
| giss_e2_r | NASA Goddard Institute for Space Studies USA |
| inm_cm4 | Russian Institute for Numerical Mathematics |
| miroc_esm | University of Tokyo, National Institute for Environmental Studies, and Japan Agency for Marine-Earth Science and Technology |
| miroc_esm_chem | University of Tokyo, National Institute for Environmental Studies, and Japan Agency for Marine-Earth Science and Technology |
| miroc_miroc5 | University of Tokyo, National Institute for Environmental Studies, and Japan Agency for Marine-Earth Science and Technology |
| mohc_hadgem2_cc | UK Met Office Hadley Centre |
| mohc_hadgem2_es | UK Met Office Hadley Centre |
| mri_cgcm3 | Meteorological Research Institute |
| ncar_ccsm4 | US National Centre for Atmospheric Research |
| ncc_noresm1_m | Norwegian Climate Centre |
| nimr_hadgem2 | UK Met Office Hadley Centre |
| Cccma_canesm2 | Canadian Centre for Climate Modelling and Analysis, Canada |
| Mpi_esm_lr | Max Planck Institute for Meteorology, Germany |
