# Supplementary figures and images for: Climate change implications for the distribution of the babesiosis and anaplasmosis tick vector, Rhipicephalus (Boophilus) microplus
Source: Vet Res. 2020 Jun 17;51:81. doi: 10.1186/s13567-020-00802-z (PMC7298856; doi:10.1186/s13567-020-00802-z)

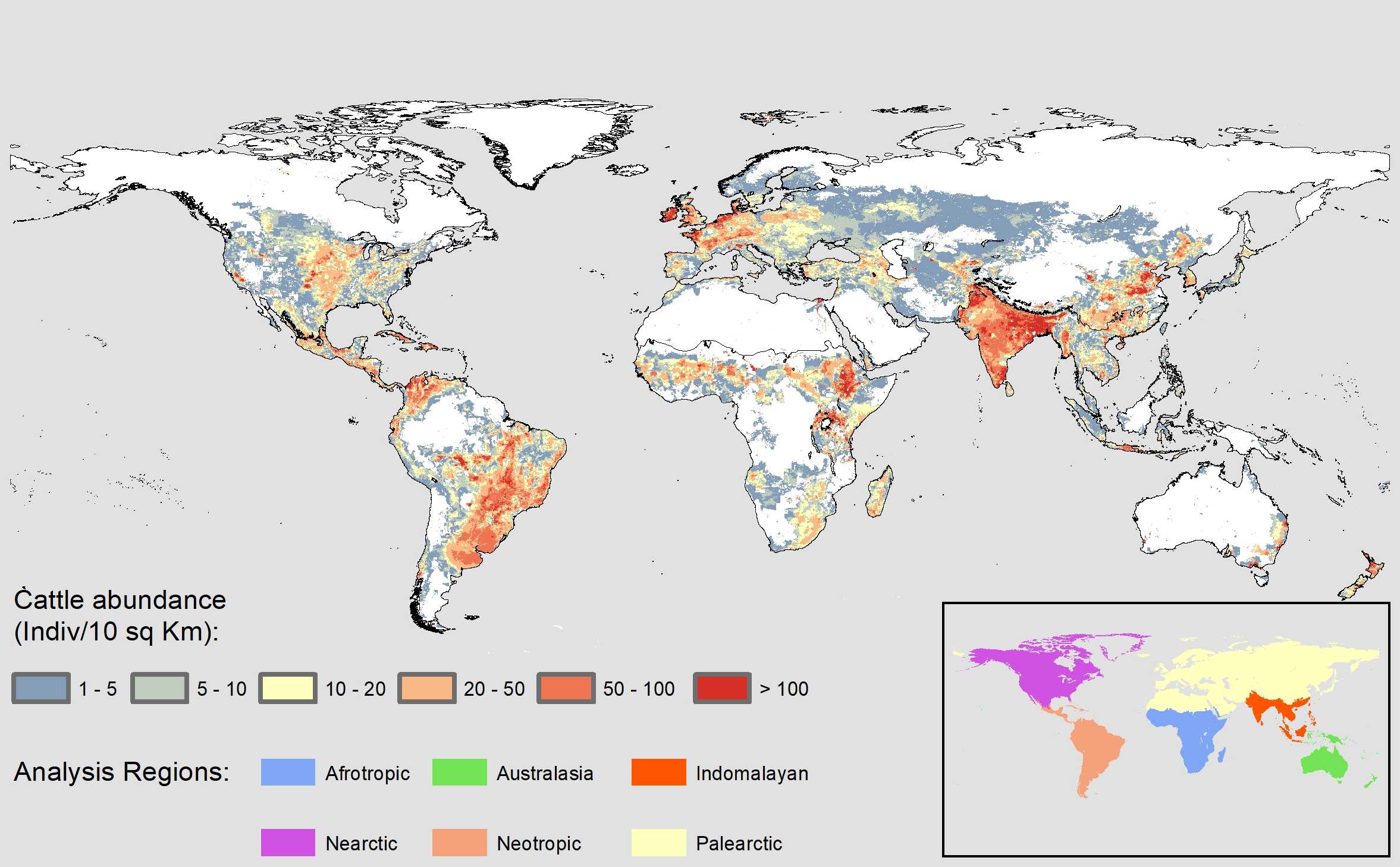

Supplement: Supplementary file 2 — Additional file 2. Cattle abundances categorized from FAO and Robinson et al. [54] from different zoogeographic regions in the world to evaluateRhipicephalus(Boophilus)microplussuitability in each of them under future climate change and present-day scenarios. Abundances are represented by number of individual heads of cattle per 10 km2. [file 13567_2020_802_MOESM2_ESM.doc]

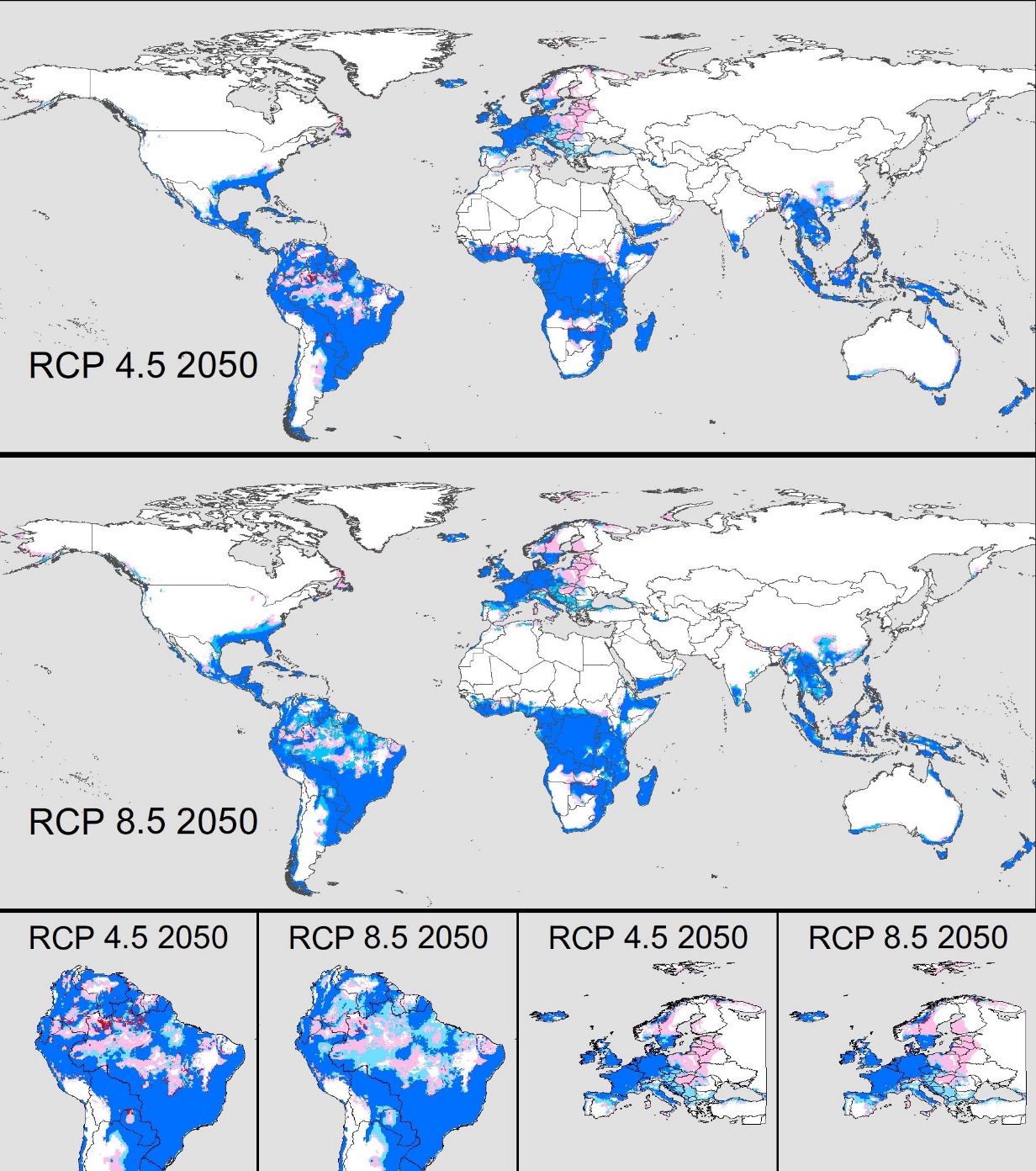

Supplement: Supplementary file 3 — Additional file 3. Current and potential future distributions ofRhipicephalus(Boophilus)microplusfor two emissions scenarios (top, RCP 4.5; bottom, RCP 8.5) in 2050. Dark blue: areas predicted to be suitable in present-day and with a strong chance of suitability in the future (> 12 GCM). Light blue: areas predicted to be suitable in present-day but with reduced probability of presence in the future (< 12 GCM). Red: areas unsuitable in the present-day, but with a strong chance of suitability in the future (> 12 GCMs). Pink: areas predicted to be unsuitable in present-day but have slight chance of suitability in the future (< 12 GCM). White: areas unsuitable in both present-day and future scenarios. [file 13567_2020_802_MOESM3_ESM.doc]

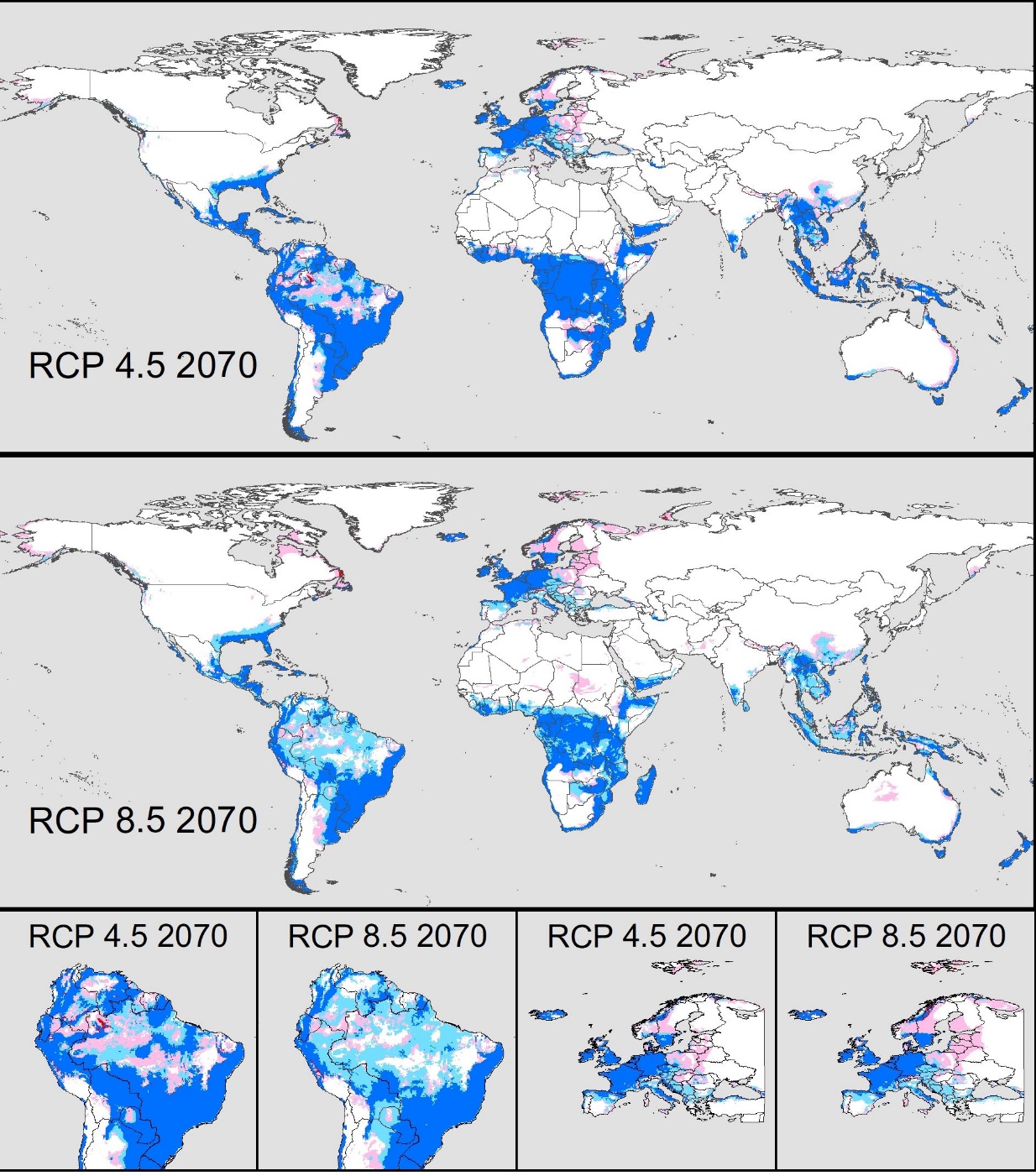

Supplement: Supplementary file 4 — Additional file 4. Current and potential future distributions ofRhipicephalus(Boophilus)microplusfor two emissions scenarios (top, RCP 4.5; bottom, RCP 8.5) in 2070. Dark blue: areas predicted to be suitable in present-day and with a strong chance of suitability in the future (> 12 GCM). Light blue: areas predicted to be suitable in present-day but with reduced probability of presence in the future (< 12 GCM). Red: areas unsuitable in the present-day, but with a strong chance of suitability in the future (> 12 GCM). Pink: areas predicted to be unsuitable in present-day but with a slight chance of suitability in the future (< 12 GCM). White: areas unsuitable in both present-day and future scenarios. [file 13567_2020_802_MOESM4_ESM.doc]

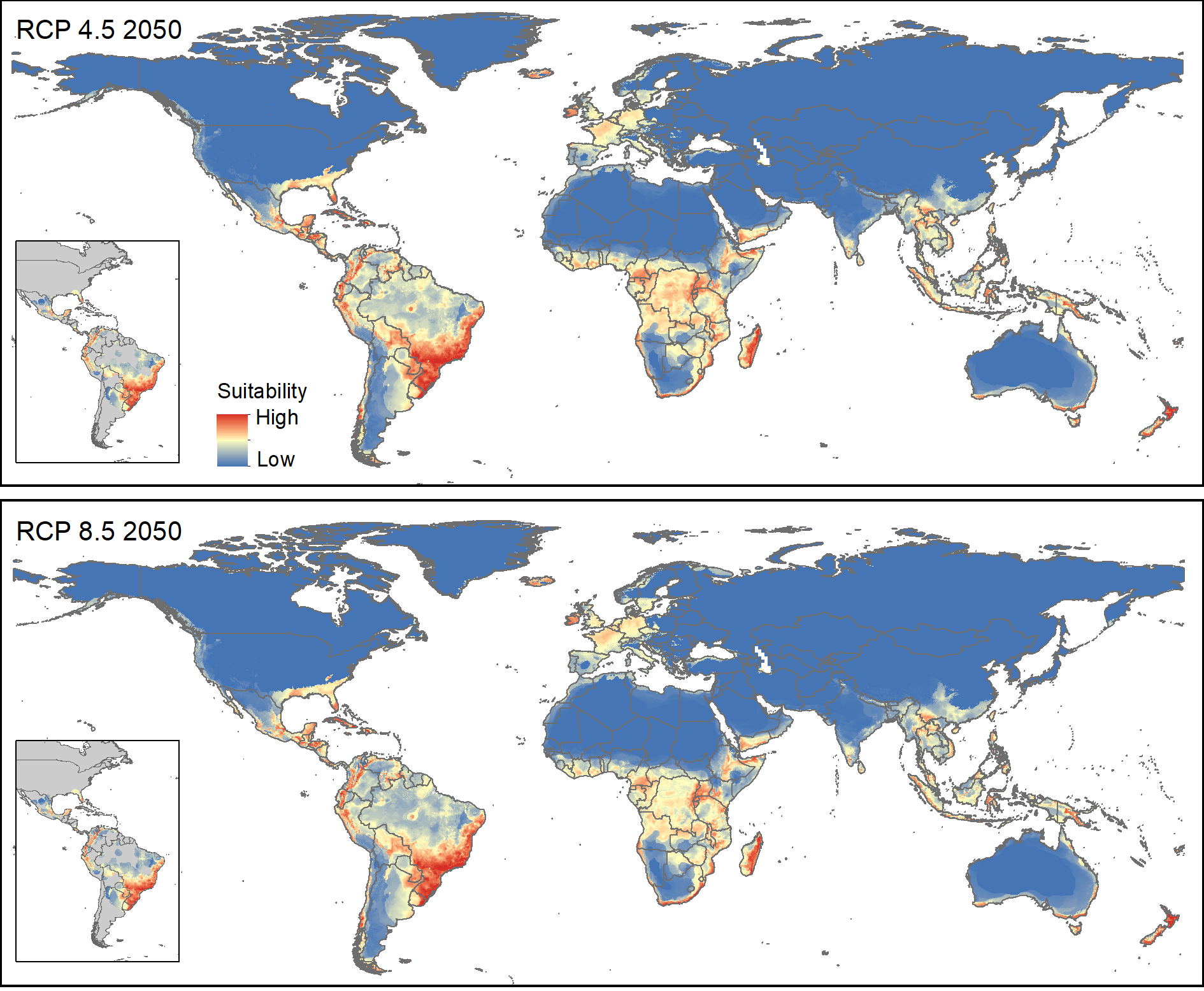

Supplement: Supplementary file 5 — Additional file 5. Suitability forRhipicephalus(Boophilus)microplusin 2050 under RCP 4.5 and 8.5 according to the best ecological niche model. [file 13567_2020_802_MOESM5_ESM.doc]

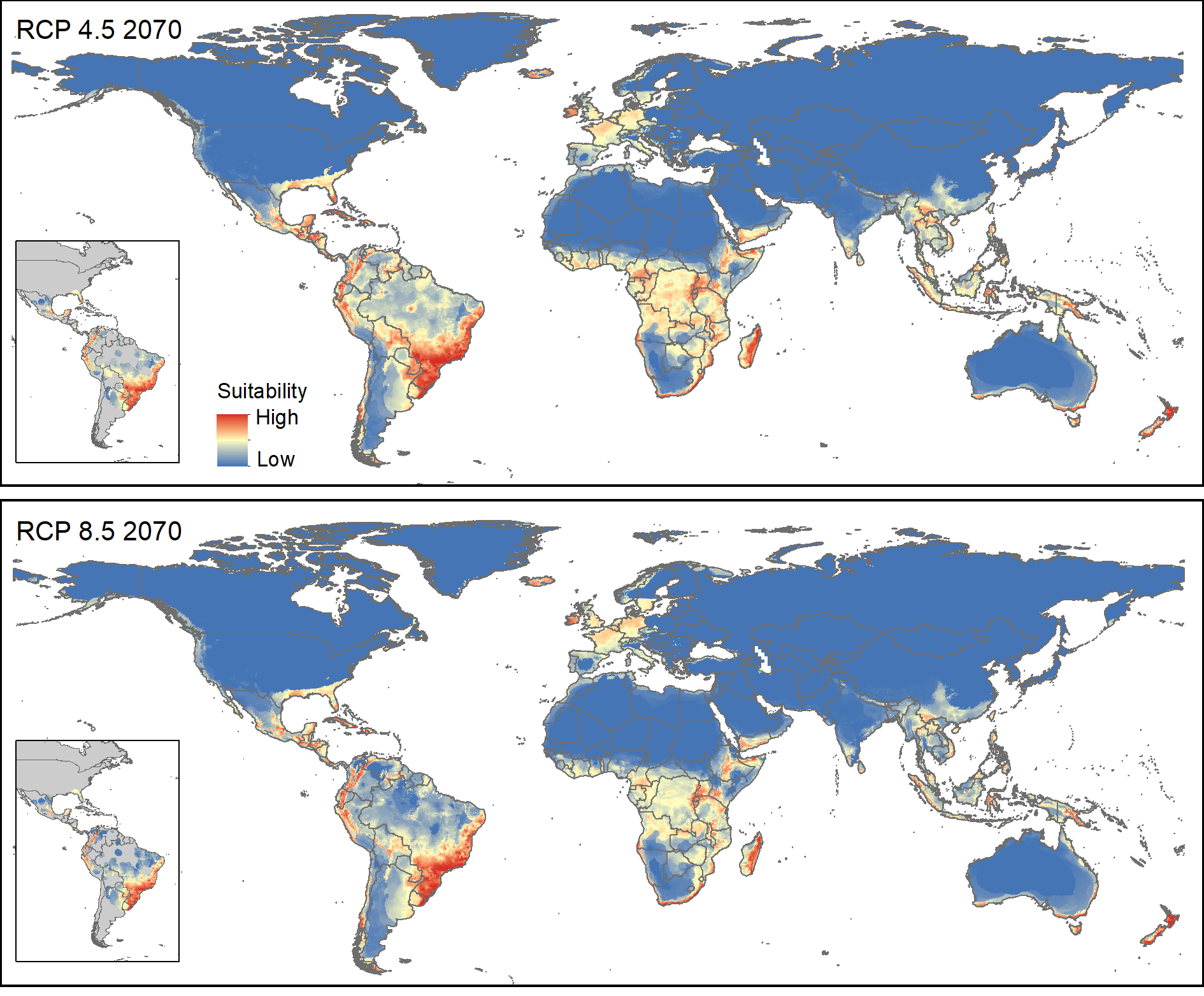

Supplement: Supplementary file 6 — Additional file 6. Suitability forRhipicephalus(Boophilus)microplusin 2070 under RCP 4.5 and 8.5 according to the best ecological niche model. [file 13567_2020_802_MOESM6_ESM.doc]
